# Assessment of occupational injuries among Addis Ababa city municipal solid waste collectors: a cross-sectional study

**DOI:** 10.1186/1471-2458-14-169

**Published:** 2014-02-17

**Authors:** Daniel Bogale, Abera kumie, Worku Tefera

**Affiliations:** 1 College of Medicine and Health Sciences, Madawalabu University, Postal address: 139 Bale Goba, Ethiopia; 2 School of Public Health, Addis Ababa University, Addis Ababa, Ethiopia

## Abstract

**Background:**

Collection of household waste is a job which requires repeated heavy physical activities such as lifting, carrying, pulling, and pushing. Like many developing countries, in Ethiopia municipal solid waste is collected manually. Therefore, this study is aimed to assess the extent of occupational injuries and associated factors among solid waste collectors in Addis Ababa City.

**Methods:**

A cross-sectional study was conducted among 876 respondents sampled from 92 unions. A pre-tested structured questionnaire and observation check list were used to collect data. Crude odds ratio with 95% CI was computed to see the presence of association between selected independent variables and occupational injury. Multivariate logistic regression analysis was made to see the relative effect of independent variable on the dependent variable by controlling the effect of other variables. To maintain stability, only variables that have a p-value less than 0.30 in the binary logistic regression analysis were kept in the subsequent model. Enter method was used hierarchically.

**Results:**

The response rate of this study was 97.9%. Female respondents accounted 71.2%. The median age of the study subjects was 33 year (with 52 inter quartile range). The overall occupational injury prevalence rate in the last 12 months was 383 (43.7%). Utilization of personal protective devices and family size in the household were statistically associated with injury. As compared to workers who used personal protective equipments while being on duty, odds of injury among workers not used personal protective equipments were 2.62 higher (AOR = 2.62, 95% CI: 1.48-4.63). As compared to those who had five and more children, odds of injuries among those who had 3-4 children was reduced by half (AOR = 0.52, 95% CI: 0.30-0.93).

**Conclusion:**

The extent of occupational injuries among Addis Ababa city solid waste collectors is present in a level that needs immediate public health action. Implementation of basic occupational health and safety services including training on occupational health and safety, ensuring the provision and use of personal protective devices are highly advisable.

## Background

Globally, solid waste collectors are exposed to occupational health related problems from waste materials and physical effort they exert in waste handling. Such occupational risk include, but not limited to contact with human faecal matter, part of waste that may have contaminated with toxic materials, bottles with chemical residues, metal containers with residue pesticides and solvents, sharps and other infectious wastes from hospitals, and batteries containing heavy metals. They are also exposed to exhaust emissions of refuse trucks [1-3]. The waste collector’s job involves repetitive motion, awkward working positions, forceful hand exertion and frequent manual handling. Dim lighting in early morning hours, and rain are inevitable. All such conditions potentially contribute to ergonomic problems [1].

Standard operation procedures in handling municipal solid wastes in industrialized countries have reduced occupational and environmental impacts significantly. However, the risk levels are still very high in developing countries because of poor public health practice. In low-income countries, solid waste collectors have low socio-economic status such as poverty, lack of education, poor housing conditions and poor nutrition. Farther more, this group of workers is exposed directly and without adequate personal protection to municipal solid waste (MSW) which includes hazardous substances [2-4]. Commonly observed health problems among this working group include respiratory symptoms, irritation of the skin, nose and eyes, gastrointestinal problems, fatigue, headaches, psychological problems, allergies, musculoskeletal and dermal injuries [5,6].

Apart from the social atrocities that workers face, they are exposed to certain health problems by virtue of their occupation [7]. In order to work, especially at physically demanding jobs such as solid waste collection, the worker must be relatively healthy. In this environment, the worker’s health is his/her greatest asset and a precondition for the sustainable generation of income. Protection of workers from occupational hazards depend on availability and proper utilization of protective equipments, which in low and middle income countries is in short supply with very limited monitoring of their utilization [8]. Moreover, refuse workers often lack training, tools and information in order to perform their work in the best healthy and safe manner. In addition to these, routine medical checkup program for all solid waste collectors is mandatory to keep them safe and secure [7,9,10].

Municipal solid waste in Addis Ababa (AA) city is collected manually which requires repeated heavy physical activities such as lifting, carrying, pulling, and pushing. The waste awaiting collection is readily available to insects and rodents and scavenging animals which are potential carriers of enteric pathogens. It also transferred from any kind of household container into sacks or directly into a pushcart which is often pushed over rough, unpaved or cobbled, inclined roads to collection sites. Then, it is manually emptied in to a bigger container having a volume of 8 m^3^ or in to a refuse truck. Workers are less protected in all efforts of refuse collection. Neither pre-employment nor periodical medical checkups are inaccessible to this group of workers.

The provision of house to house refuse collection service is a recent practice in AA. Occupational related studies are lacking given that the possible exposure of workers to various work related hazards might exist. Information to policy makers to improve the working condition as a result is limited. Therefore, this study was designed to investigate the magnitude of occupational injuries and contributing factors among solid waste collectors in AA city.

## Methods

### Study design and setting

This study employed a quantitative cross-sectional study design. The study was carried out in Addis Ababa, capital city of Ethiopia. The city has a total of 2,738,248 population with an area of 540 square kilometers (54000 hectares) and it is sub-divided in to ten sub-cities.

From the total waste generated in the city of Addis Ababa, 1482 m^3^ of waste is collected and transported to disposal site per day and 540,789 m^3^ per annual. Currently a number of micro and small enterprises (MSE) are emerging to participate in primary solid waste collection. The emerging MSEs collect household refuse and transfer to the municipal waste containers and transfer points [11]. During this study, the number of enterprises was 518 with a total number of 5454 operators.

### Population

Solid waste collectors working throughout the city were the source population where as workers in Addis Ketema, Kirkos, Lideta, Nifas Silk Lafto and Kolfe sub-cities were study population. Sampling units were household solid waste collector unions found in five sub-cities mentioned above. All workers in the selected unions who have a minimum of one year work experience were included in the study.

### Sample size determination

This study has two objectives. In the first round, sample size was computed for both objectives separately. Then, the largest sample size was taken from the objective that yields the maximum sample size and calculated based on the following assumptions:

Proportion of occupational injuries among Personal Protective Equipment (PPE) users was assumed to be 50%. Assuming a minimum of 10% difference detection rate of injury among PPE users and non users and Odds of occupational injuries among non users was assumed 1.5 times higher than PPE users [12]. Finally, these values were entered in to Epi Info version 3.5.1 software.

### Sampling procedures

A two-stage cluster sampling method was used to select 50% of sub-cities and about one third of unions in the selected sub-cities. Ninety unions from the five sampled sub-cities were selected proportionally to the number of unions they have. Unions allocated to each sub-city were randomly selected from the list of total unions in that sub-city. Finally all workers in the selected union were interviewed.

### Data collection tool and procedures

Structured questionnaire was adopted from ILO Occupational injury statistics and different relevant sources [13-16] with required modification based on research objectives. There was also observation checklist for Personal Protective Devices availability and utilization observed for each worker on duty. The questionnaire was prepared in English and then translated to Amharic and retranslated back to English to insure its consistency. Pretest was conducted 15 days prior to actual data collection on one sub-city which was not included in the actual study for validation of data collection tool. Five bachelor science degree holders collected the data from workers on duty during data collection period.

### Operational definition

#### Injury

The reported work related physical damage to body tissues caused by accident or by exposure to environmental stressor in the last one year prior this data collection [17].

#### Job satisfaction

It is a subjective response of study participants about their job as it is pleasurable for them.

#### Micro and Small Enterprise (MSE)

Small scale unions that are organized by government to collect waste from households to specific site that is accessible for transportation to final disposal site.

#### Well dressed

If the workers are wearing protective devices properly on the right body parts for that particular PPE while they are on duty at the time of observation.

#### Perforated

Devices or sacks that has porous and tear out so that it allows dust and fluid to workers body.

### Data processing and management

Coded data was organized and entered in to Epi Info version 3.5.1 computer software package. Cleaning was made to avoid missing values, outliers and other inconsistencies. Commands such as frequency, sort, find and list were used to clean the data. Cleaned data was exported to SPSS 16.0 version computer software package for analysis.

### Data analysis

Frequencies, percentages and medians of variables were computed to describe the data. Crude odds ratio with 95% CI was computed to see the presence of association between independent variables and occupational injury. Multivariate logistic regression analysis was made to observe the relative effect of independent variable on the dependent variable by controlling the effect of other variables. The multivariate logistic regression analysis was done using enter method hierarchically to assess the relative effect of the explanatory factors on the occupational injury. To limit many variables and unstable estimates in the subsequent models, only variables that reached a p-value less than 0.30 at the bivariate analysis level were kept in the subsequent model [18-22].

### Data quality assurance

To maintain the quality of the data, structured and pre-tested questionnaire was used to collect information. Two days training was given to all data collectors, and supervisors in accordance with training manual developed beforehand.

### Ethical Considerations

Ethical clearance was obtained from Institutional Review Board of College of Health Sciences, Addis Ababa University. Formal letter was written to Addis Ababa City Solid Waste Management and Recycling Project Office. The information sheet and consent form was provided for respondents to read for those who can read and for those who cannot read, interviewer read the paper. Finally, he or she was asked for his or her willingness to participate in the study. Confidentiality was maintained by omitting respondents’ name and personal identification.

## Results

### Socio-demographic characteristics of respondents

Eight hundred seventy-six municipal solid waste collectors participated in the study yielding 97.9% response rate. The majority of respondents were females which account 71.2% and the median age of respondents was 33 year with 18 and 70 minimum and maximum ages respectively. The median monthly income for the survey respondents was 400 Ethiopian birr with a range of 200 to 900 birr. Five hundred twenty three (59.7%) of the respondents were working through all days of the week. Eight hundred forty-two (96.1%) of study participants were working 8 hours and below per a day. Six hundred twenty four (71.2%) study participants were female (see Table 1).

**Table 1:** Respondents by socio- demographic characteristics of AA city solid waste collectors, January 2012

**Variables**	**Frequency (n = 876)**	**Percent**
**Sex**		
Male	252	28.8
Female	624	71.2
**Marital status**		
Married	512	58.4
Single	191	21.8
Divorced/Separated	90	10.3
Widowed	83	9.5
**Educational level**		
Illiterate	334	38.1
Primary school	481	54.9
Secondary school and above	61	7.0
**Employment condition**		
Permanent	864	98.6
Contract	12	1.4
**Other job**		
Yes	94	10.7
No	782	89.3
**Family size**		
Two and less family	277	31.6
Three to four family	410	46.8
Five and above	189	21.6
**Work experience as waste collection**		
≤5 years	492	56.2
≥6 years	384	43.8
**Working hours per day**		
≤8 hours	842	96.1
>8 hours	34	3.9

### Occupational safety and behavioral factors

Only 382 (43.6%) of respondents used some kinds of personal protective equipments (PPE) while they are on duty. Out of these PPE users, 86 (22.5%) of them reported that they were not using it all the time while they are on duty. The main reasons mentioned by the respondents for none use of PPE were; no access 72 (83.7%), discomfort 22 (25.6%) and to save time 11 (12.8%). The majority of PPE users, 281 (73.6%) purchase PPEs for themselves and others were supplied from woreda municipality, NGOs and some picked PPE like glove from health care wastes. Only 19 (2.2%) study participants were vaccinated for infectious diseases that are risk for cleaning workers. See Table 2 for occupational safety and behavioral variables.

**Table 2:** Utilization of PPE and behavioral status of AA city solid waste collectors, January 2012

**Variables**	**Frequency (n = 876)**	**Percent**
**PPE on duty**		
Yes	382	43.6
No	494	56.4
**PPE all the time**		
Yes	296	77.5
No	86	22.5
**First training**		
Yes	182	20.8
No	694	79.2
**On job training**		
Yes	502	57.3
No	374	42.7
**Smoking cigarette**		
Yes	78	8.9
No	798	91.1
**Drinking alcohol**		
Yes	77	8.8
No	799	91.2
**Chewing chat**		
Yes	106	12.1
No	770	87.9
**Sleeping disorder**		
Yes	121	13.8
No	755	86.2
**Job satisfaction**		
Yes	789	90.1
No	87	9.9
**Vaccinated for Tetanus**		
Yes	19	2.2
No	857	97.8

*PPE* Personal Protective Equipments.

*First training* means whether the study participants were trained about safety issues first when they engaged in the current line of job or not.

**Figure 1:**
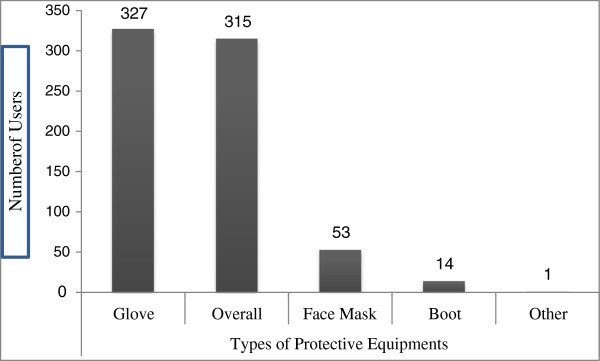
**Types of personal protective equipments used by Addis Ababa city solid waste collectors, January 2012.** Note: On Figure 1, the sum of PPE users exceeded 382 because a respondent might use more than one type of personal protective equipments.

The common types of protective devices utilized by the study participants were assessed. For the types of protective materials used by the study subjects, see Figure 1.

### Occupational injury

The overall prevalence of occupational injury was 43.7% (95% CI: 40.7, 47.1). Hands were the most injured body parts and cut was the common injury type. See Table 3.

**Table 3:** Injured body parts and injury types during last 12 months AA city SW collectors, January 2012

**Variables**	**Frequency**	**Percent**
**Occupational injuries**		
In the past 12 months (n = 876)	383	43.7
**Occupational injuries**		
In the past one month (n = 383)	243	27.7
**Number of occurrence** (n = 243)		
Once	52	21.4
Twice	101	41.6
More than two times	90	37.0
**Injured body parts** (n = 383)		
Hand	232	60.75
Finger	89	23.32
Leg	79	20.63
Back	44	11.49
Knee	39	10.18
Toe	15	3.92
Eye	9	2.35
Tooth	8	2.09
Head	8	2.09
***Types of injury (n = 383)***		
Cut	221	57.70
Puncture	146	38.12
Fall	76	19.84
Abrasion	45	11.75
Fracture	18	4.70
Strain	13	3.40
Dislocation	4	1.04
Burn	2	0.52
Other*	12	3.13

*Other includes: dog bite, chemical splash, car and bicycle accident and snake bite.

In the rows indicating injured body parts and types of injuries the sum of percents exceeded 100 because it has multiple responses.

**Figure 2:**
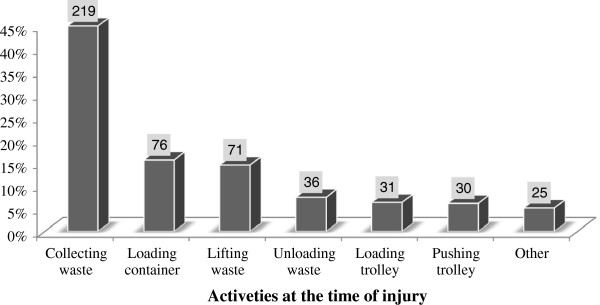
**Types of work performed when injury happened among AA City solid waste collectors, January 2012.** Other includes loading the track and sweeping around the container at the curb side. It had multiple responses.

Workers were exposed for injuries while they were on duty. Some of these works were transferring wastes from household’s container and picking openly disposed wastes from the ground. Figure 2 indicates types of activities workers perform when injury was occurred. See Figure 2.

### Bivariate analysis

The association between socio-demographic variables and occupational injuries was computed. As the age of solid waste collectors increased by one year, odds of occupational injury was also increased by 3% (COR = 1.03, 95%CI: 1.01-1.04). Number of working hours per day was significantly (COR = 1.18, 95% CI: 1.03-1.34) associated with occupational injury. Work experience was another variable that is significantly associated with occupational injury (COR = 1.08, 95% CI: 1.03-1.13). Smoking cigarette (COR = 2.10, 95% CI: 1.30-3.40) and drinking alcohol (COR = 1.92, 95% CI: 1.19-3.08) were the significant behavioral variables to injury in the bivariate analysis. Similarly, sleeping disorder (COR = 1.72, 95% CI: 1.17-2.54) was other statistically significant variable.

### Multivariate analysis

In the first step, the effects of selected socio-demographic variables on occupational injuries were assessed. In the second step of the analysis, occupational safety variables were added, and their effect was assessed in the presence of socio-demographic variables that had p value < 0.3. Behavioral factors were entered in third step. In this step, the effect of the selected behavioral factors was assessed in the presence of both socio-demographic and occupational safety variables that had p-value < 0.3.

From all variables entered in all steps of analysis, only family size and utilization of PPE all the time while on duty were remained significant after adjusting for other socio-demographic, occupational safety and behavioral factors. See Table 4.

**Table 4:** Logistic regression analysis result on occupational injuries among AA city MSW collectors, January 2012

**Characteristics**	**Crude OR (95% CI)**	**Adjusted OR (95% CI)**		
		**Model 1**	**Model 2**	**Final model**
*Model 1*: **(socio-demographic variables)**				
**Sex**				
(Male Vs Female^RG^)	1.40 (1.04–1.87)*	2.35 (1.65–3.36)**	1.55 (0.80–3.04)	
**Educational status**				
(Illiterate Vs ≥2^0 RG^) (1^0^ school Vs ≥2^0 RG^)	2.20 (1.22–3.92)* 1.34 (0.76–2.35)	2.35 (1.21–4.54)* 1.41 (0.78–2.56)		
**Family size**				
(Less or 2 Vs ≥5^RG^) (3–4 Vs ≥5^RG^)	0.50 (0.35–0.73)** 0.72 (0.51–1.01)	0.67 (0.44–1.04) 0.86 (0.60–1.24)	0.24 (0.11–0.51)** 0.54 (0.30–0.98)*	0.21 (0.10–0.44)** 0.52 (0.30–0.93)*
**Experience**				
(≤5 yrs^RG^ Vs ≥6 yrs)	1.50 (1.14–1.95)*	1.20 (0.85–1.69)		
**Daily work hours**				
(≤ 8 hrs^RG^ Vs > 8 hrs)	2.80 (1.35–5.82)*	2.67 (1.26–5.70)*	2.55 (0.68–9.56)	
*Model 2*: **(socio–demographic variables + 0ccupational safety variables)**				
**PPE on duty**				
(Yes^RG^ Vs No)	4.13 (3.08–5.53)**		––––––––––––––––	––––––––––––––––––
**PPE all the time**				
(Yes^RG ^Vs No)	2.41 (1.44–4.03)**		2.61 (1.48–4.59)*	2.62 (1.48–4.63)*
**First training**				
(Yes^RG^ Vs No)	0.74 (0.53–1.03)		0.98 (0.56–1.72)	
**On job training**				
(Yes^RG^ Vs No)	1.45 (1.10–1.91)**		1.05 (0.60–1.85)	
*Model 3*: **(Socio–demographic variables + Occupational safety variables + Behavioral variables)**				
**Smoking cigarette**				
(Yes Vs No^RG^)	2.10 (1.30–3.40)*			1.69 (0.48–6.00)
**Chewing chat**				
(Yes Vs No^RG^)	1.33 (0.88-2.00)			
**Drinking alcohol**				
(Yes Vs No^RG^)	1.92 (1.19–3.08)*			1.48 (0.49–4.47)
**Sleeping problem**				
(Yes Vs No^RG^)	1.72 (1.17–2.54)*			1.64 (0.77–3.46)
**Job satisfaction**				
(Yes^RG^ Vs No)	1.11 (0.71–1.73)			0.77 (0.34–1.77)

*RG* Reference Group. **Significant at p-value < 0.01, *Significant at p-value < 0.05.

### Findings from observation

#### Personal Protective equipments availability and utilization (clothing)

Out of 327 observed gloves on workers on duty, 117 (35.8%), 199 (60.8%), 208 (63.6%) and 129 (39.4%) were new, water proof, well dressed and perforated respectively. Out of the total observed face mask 11 (20.7%) was perforated. Out of 315 overall clothing observed on workers on duty, 208 (66.0%) well dressed, 90 (28.6%) perforated, 103 (32.7%) new and 53 (16.8%) water proof. Fourteen (1.6%) workers used boot where as others used short shoes, sleeper, and others were bare footed while they were on duty.

#### Types and status of cart and sacks

Types of carts were observed whether it is made from wood or metal which might be a cause for occupational hazards. The majority of carts were made from metal which accounts 92.5% where as the rest were made up of wood. Three hundred sixty five of observed carts were easily movable by waste collectors and the rest, 302 were not easily movable either due to their size or the types of the wheel they have. These carts were further observed for the smoothness of their hand and general body parts that might challenge pushing and empting activities. Of which 284 were rough which might be an ergonomic hazards for different body parts. The statuses of 867 waste collection sacks were observed on the spot; 852 were old, 604 were perforated and 833 were over filled.

## Discussion

The response rate of this study was 97.9% that seems higher than previous similar studies, 92% [13] and 95% [14]. This could be resulted from the effort made to minimize the non response rate by repeatedly visiting the workers. Number of female workers was higher as compared with some other studies those either with no or small number of female workers in this sector [21,23]. The main reason for large number of females workers in this study might be this work sector is an emerging and leveled as one of small scale enterprises in the country and females are actively involved in the sector [24].

The overall prevalence rate of work related injury within the past 12 months was 383 (43.7%), which is comparable with Colombo Municipal Council workers but higher as compared with study done in Alaska [12,25]. This difference might be due to variation in regulation and culture of the residents on waste segregation at house hold level or the pattern of PPE utilization by collectors across different countries. The magnitude of injuries in this study was further compared with studies conducted in other sectors like different scale industries and farming sectors that measured the rate of injuries within 12 months. It was higher than the prevalence of work related injury on small and medium scale industry that was 33.5% [13] but it was lower than injury rates on large scale metal manufacturing industry and Tendaho agricultural development sector which were 48.9% and 78.3% respectively [11,14]. This discrepancy could be resulted from the variation and nature of activities performed at different work sectors.

In this study waste collection had the highest incidents resulting in injury which was 219 (44.9%) followed by loading and lifting 79 (15.6%) and 71 (14.6%) respectively. These activities were also had the highest incident in other study done in Alaska [25]. The possible explanation for this could be manually loading waste in to sacks, pushing and pulling through long distance to be loaded in to storage containers might increase exposure for injuries. The main reported occupational injury types were cut, 57.7% and puncture, 38.1% that is slightly lower than finding in Colombo, cut 74.4% and needle/nail prick 42.5% [12]. This slight difference could be due variation in waste composition. Hands are the most commonly injured body part followed by finger which is consistent with the study conducted among cleaners in Germany [9]. Similarly, these two body parts were the first to be injured in other occupational sectors [11,14]. This might be due to the fact that waste collectors wipe waste and put it in to the cart and tracks using their feet or hands which increase the probability of cut, bruises and ruptures.

The absence of safety training, especially on job training, limited use of personal protective devices while on duty and prolonged duration of working hours were major factors that contribute to the occurrence of injury. Those who were not using PPE all the time while on duty had 2.62 times higher occupational injury than those who use PPE all the time while on duty. This is consistent with the findings from other settings [4,18]. The risk of occupational injury for those who had two or less children was reduced by 79% as compared with those who had five or more children (AOR = 0.21, 95%CI: 0.10-0.44). Similarly, odds of injuries for those who had 3-4 children were reduced by half (AOR = 0.52, 95%CI: 0.30-0.93). The possible explanation for this could be respondents who had more children might be pre-occupied by extra thinking about their children which might increase the risk of occupational injuries among this group. Another possible reason could be those who had large family size might not afford to buy PPE and use it consistently.

Work-related musculoskeletal disorders (MSDs) are impairments of bodily structures such as muscles, joints, tendons, ligaments, bones and the localized blood circulation system, that are caused or aggravated primarily by work and by the effects of the immediate environment in which work is carried out. In this study total of 311 (35.5%) participants reported that they had been troubled with musculoskeletal symptoms (joint and back pain) during the last 12 months which is nearly similar figure with study in Colombo, 38.3% [12]. However, it is not comparable with study among Tehran solid waste workers 142 (65%) [10]. This difference might be due to knowledge of participants in reporting the problem or the difference in designing data collection instrument. This huge magnitude is obviously resulted from the nature of the work which needs repeated heavy physical work such as lifting, carrying, pulling, and pushing.

This study tried to reduce recall bias by asking occurrence of occupational injury in the last twelve months and one month prior this data collection. However, it is impossible to avoid recall bias totally. In addition to this, cause and effect relationship might not be established due to the cross sectional nature of the study.

## Conclusion

The extent of occupational injuries among Addis Ababa city solid waste collectors is present in a level that needs immediate public health action. Personal protective equipment utilization is the determinant factor for occupational injuries that arise in this sector. Therefore, implementation of basic occupational health and safety services including the provision of personal protective devices and insuring utilization are highly advisable.

## Competing interests

The authors declare that they have no competing interests.

## Authors’ contributions

DB was the principal investigator of the study and took the leading role from conception, design and supervising data collection process to the final analysis and preparation of the manuscript. AK participated in the design of the study and reviewing the whole document especially on the method part. WT participated in reviewing the method part and provided critical comments. All authors read and approved the final manuscript.

## Pre-publication history

The pre-publication history for this paper can be accessed here:

http://www.biomedcentral.com/1471-2458/14/169/prepub
